# Altered Monocyte and Langerhans Cell Innate Immunity in Patients With Recurrent Respiratory Papillomatosis (RRP)

**DOI:** 10.3389/fimmu.2020.00336

**Published:** 2020-03-10

**Authors:** Mohd Israr, James A. DeVoti, Fung Lam, Allan L. Abramson, Bettie M. Steinberg, Vincent R. Bonagura

**Affiliations:** ^1^Barbara and Donald Zucker School of Medicine at Hofstra/Northwell, Feinstein Institutes for Medical Research, Manhasset, NY, United States; ^2^Department of Otolaryngology, Long Island Jewish Medical Center, Barbara and Donald Zucker School of Medicine at Hofstra/Northwell, New Hyde Park, NY, United States; ^3^Department of Pediatrics, Steven and Alexandra Cohen Children's Medical Center of New York, Barbara and Donald Zucker School of Medicine at Hofstra/Northwell, New Hyde Park, NY, United States

**Keywords:** recurrent respiratory papillomatosis, human papillomaviruses (HPVs) types 6 and type 11, Langerhans cell, monocyte, innate immunity, PGE_2_

## Abstract

The micromilieu within respiratory papillomas supports persistent human papillomavirus (HPV) infection and disease recurrence in patients with recurrent respiratory papillomatosis (RRP). These patients show polarized (T_H_2-/Treg) adaptive immunity in papillomas and blood, enriched immature Langerhans cell (iLC) numbers, and overexpression of cyclooxygenase-2/prostaglandin E_2_ (PGE_2_) in the upper airway. Blood monocyte-derived, and tissue-derived iLCs from RRP patients and controls were now studied to more fully understand innate immune dysregulation in RRP. Patients' monocytes generated fewer iLCs than controls, due to a reduced fraction of classical monocytes that generated most but not all the iLCs. Prostaglandin E_2_, which was elevated in RRP plasma, reduced monocyte-iLC differentiation from controls to the levels of RRP patients, but had no effect on subsequent iLC maturation. Cytokine/chemokine responses by iLCs from papillomas, foreskin, and abdominal skin differed significantly. Freshly derived tissue iLCs expressed low *CCL-1* and high *CCL-20* mRNAs and were unresponsive to IL-36γ stimulation. Papilloma iLCs uniquely expressed IL-36γ at baseline and expressed *CCL1* when cultured overnight outside their immunosuppressive microenvironment without additional stimulation. We conclude that monocyte/iLC innate immunity is impaired in RRP, in part due to increased PGE_2_ exposure *in vivo*. The immunosuppressive papilloma microenvironment likely alters iLC responses, and vice versa, supporting T_H_2-like/Treg HPV-specific adaptive immunity in RRP.

## Introduction

Recurrent respiratory papillomatosis (RRP), characterized by the recurrent growth of premalignant airway lesions, is a rare disorder predominantly caused by human papillomaviruses (HPVs) types 6 and type 11 (1). Patients with RRP can present with a mild course of disease that requires infrequent surgical removal that may vary in time between surgeries to remove these premalignant tumors interspersed with periods of clinical remission. With more severe disease (rapid regrowth of papillomas or progression to involve the trachea, bronchi, or lungs), frequent surgical removal in excess of three times per year may be required to maintain a patent airway (2). Recurrent respiratory papillomatosis causes significant morbidity and, on occasion, mortality, because of the location of these lesions in the upper airway and the high rate of conversion to squamous cell carcinoma when there is pulmonary involvement (3). Recurrence of papillomas is caused by activation of latent HPV infection that is widespread throughout the airway (2, 4). However, 5% of healthy individuals have latent HPV6/11 infection in their upper airway without any evidence of clinical disease (5).

Previously, we reported that patients with RRP show a T_H_2-/Treg–polarized adaptive immune response to HPV6/11, and a T_H_2-like innate response, in their respiratory papillomas and blood (6–8). However, these patients have normal immune responses to all other infectious microbes. We also identified a repertoire of immunologically relevant, adaptive and innate response genes, and the pathways that likely define these skewed responses (9). Of interest was the paradoxical observation that mRNA for the proinflammatory cytokine IL-36γ is markedly upregulated in respiratory papilloma tissues (9), without evidence of inflammation in these lesions. Furthermore, the mRNA for CCL20, which recruits regulatory T cells (Tregs) into tumors (10), is elevated, with concomitant downregulation of the proinflammatory chemokines CCL19 and CCL21 (9). The reverse is found in adjacent, clinically normal laryngeal tissues from the same RRP patients. This suggests that the balance of proinflammatory vs. anti-inflammatory immune mediators and cells within the papilloma microenvironment is altered to support persistence of HPV6/11 infection, following activation of latent infection, with subsequent papilloma recurrence (8, 11–15). We previously showed that T-cell responses to autologous papilloma tissue and to HPV6/11 early proteins are polarized away from effective viral control (8, 11, 14), likely secondary to functional PD1^+^/CD4^+^ Tregs in these lesions (15). In addition, CD8^+^/CD28^−^ T cells that express granzyme are present in papillomas but fail to function as mature cytotoxic T lymphocytes (CTLs) (8).

We have begun to explore the innate immune responses that we suspected were the cause of the skewed HPV-specific, adaptive immunity in RRP. Natural killer cells in the blood of patients with RRP, are increased, but inefficiently lyse class I major histocompatibility complex–deficient cells (8, 13). Curiously, the density of immature Langerhans cells (iLCs) is markedly increased within respiratory papillomas as compared to normal respiratory epithelial tissues (8). Langerhans cells (LCs) are a migratory group of dendritic cells (DCs) that specialize in uptake, processing, and transport of antigen and ultimately presentation of these antigens to T cells directly or via myeloid langerin^+^ DCs that can cross-present antigen to T cells (16–19). The cytokines and chemokines made by keratinocytes, as well as those made by LCs themselves, are critical in influencing LC migration and maturation, and they establish a balance that polarizes adaptive immunity toward either T_H_1 or T_H_2 responses (20–23). One possibility for this altered balance in RRP is the constitutive overexpression of cyclooxygenase-2 (COX-2) by both respiratory papillomas and clinically normal upper airway mucosal tissues from these patients (24). The overexpression of COX-2 leads to elevation of COX-2–depended prostanoids, including immunologically active PGE_2_ (25, 26).

To determine why iLCs in papillomas fail to mature (27) and to better understand the immune alteration of iLCs in RRP, we studied blood monocytes, iLCs derived from these cells, and iLCs from papillomas, foreskin, and abdominal tissue. In this communication, we describe the general characteristics of monocytes from these patients as compared to those from individuals without this disease. We examined the ability of monocytes to differentiate into iLCs and the effect of PGE_2_ on this differentiation and on the maturation of the iLCs in response to proinflammatory stimuli, including IL-36γ that is secreted in limited amounts by HPV-infected keratinocytes (28). Finally, we isolated iLCs from papilloma tissues and other epithelia to identify their expression of select cytokines and chemokines initially, in culture after removal from their microenvironment, and after proinflammatory stimulation.

## Materials and Methods

### Human Subjects

This study was conducted with blood and biopsy samples obtained from patients with RRP, blood from control individuals without RRP, and with control neonatal foreskin and adult bariatric abdominal skin from surgical discard tissues obtained at the Long Island Jewish Medical Center, Northwell Health. The studies were approved by the Northwell Health Institutional Review Board.

### Demographics, Incidence, and Disease Severity of Patients With Recurrent Respiratory Papillomatosis

The demographics of the patients with RRP used in these experiments are shown in Supplementary Table 1. Recurrent respiratory papillomatosis is a rare disease with an estimated prevalence in the United States of 4.3/10^5^ in children younger than 14 years, and of 1.8/10^5^ in adults (29, 30). The severity of RRP can be divided into two categories, severe and mild–moderate, based on the extent of disease at the time of surgery and the frequency of recurrence. At each surgery, the number of disease sites, the anatomic surface area of disease, and the extent of luminal obstruction are documented to yield a composite score as described previously (8, 31). This composite score is divided by the number of days that had elapsed since the previous surgery to yield a growth rate, which is a measurement of disease severity. The mean growth rate from multiple surgeries is used to define the overall severity score for an individual patient. An overall disease severity score of ≥0.06, or the presence of tracheal extension, is defined as severe disease. An overall severity score of <0.06 and the absence of tracheal extension are defined as mild–moderate disease.

### Measurement of PGE_2_ Plasma Levels

Whole blood was collected in 10 mL heparin-containing Vacutainer tubes and centrifuged at 1,200 *g* for 10 min at room temperature to separate cells from the plasma. Aliquots of plasma were collected and immediately frozen at −70°C until use. A forward sequential competitive binding enzyme-linked immunosorbent assay (ELISA) (PKGE004B; R&D Systems, Minneapolis, MN, USA) was used as per the manufacturer's specifications. The cross reaction of anti-PGE_2_ antibodies in this kit with other prostenoids is <5% as per the manufacturer. All samples were run in triplicate, and mean plasma levels ± SD calculated.

### Isolation, Culture, and Characterization of Total Monocyte-Derived Langerhans Cells

Peripheral blood mononuclear cells (PBMCs) were isolated from heparinized blood using Ficoll-Hypaque density gradient centrifugation (GE Healthcare, Fairfield, CT, USA). Monocytes were negatively selected using a commercially available magnetic isolation kit (Pan Monocyte Isolation Kit; Miltenyi Biotec, Bergisch Gladbach, Germany), which consistently yielded >90% purity. To generate iLCs, monocytes were cultured for 7 days in complete medium, which consisted of RPMI 1640 medium (Gibco/Life Technologies [Thermo Fisher Scientific Inc., Walthem, MA, USA]), 2 mM l-glutamine, 10 mg of streptomycin, 10,000 U/mL of penicillin G, and 10% fetal bovine serum (FBS) (Atlanta Biologicals, Flowery Branch, GA, USA), supplemented with 100 ng/mL granulocyte-macrophage colony-stimulating factor (GMCSF), 20 ng/mL IL-4, and 10 ng/mL transforming growth factor β1 (TGFβ1) (R&D Systems) (27). Cells were supplemented with complete medium + GMCSF and TGFβ1, but without IL-4, on days 2 and 4. On day 7, cells were collected, and iLCs were positively selected using CD1a MicroBeads (Miltenyi Biotec). Immature LCs were stained for viability (Live/Dead Fixable Aqua Dead Cell Stain Kit (Invitrogen/Life Technologies, Thermo Fisher Scientific Inc.) and then surface stained with fluorochrome-labeled antibodies specific for CD1a–fluorescein isothiocyanate (FITC) (BD Biosciences, San Jose, CA, USA) and E-cadherin-APC (Bio Legend, San Diego, CA, USA) and analyzed on a BD FACS Canto II (BD Biosciences). To determine the effect of PGE_2_ on generation of monocyte-derived iLCs, 250 nM PGE_2_ was added for all 7 days, and the iLCs were further selected for expression of CD207 (langerin) (anti–CD207-PE).

### Isolation of Monocyte Subpopulations and Differentiation Into iLCs

Peripheral blood mononuclear cells were isolated and monocytes purified by negative selection as stated above. Monocytes were washed once in phosphate-buffered saline (PBS) and immunostained with anti–CD14-PE and anti–CD16-Pacific Blue (BD Biosciences). Doublets and dead cells were excluded; monocytes were selected based on their light scatter and CD14 and CD16 expression and sorted into CD14^++^ CD16^−^ (classical) CD14^++^ CD16^+^ (intermediate) and CD14^−^ CD16^++^ (non-classical) monocytes using a BD FACSAria sorter (BD Biosciences), and the percentage of monocytes in each subpopulation calculated. All three subpopulations of monocytes were cultured for 7 days in complete RPMI medium supplemented with 100 ng/mL GMCSF, 20 ng/mL IL-4, and 10 ng/mL TGFβ1 as above. On day 7, cells were harvested, surface stained with fluorochrome-labeled antibodies CD1a-FITC and E-cadherin-APC, and then analyzed as above on a BD FACS Canto II (BD Biosciences).

### *In vitro* Maturation of Monocyte-Derived iLCs

Purified iLCs, generated as above and selected with CD1a^+^ microbeads, were cultured for 48 h in complete RPMI medium with either 10 ng/mL of recombinant active IL-36γ (a.a.18–169) (R&D Systems), 250 nM PGE_2_ + 10 ng/mL IL-36γ, or 100 ng/mL lipopolysaccharide (LPS) (Sigma-Aldrich, St. Louis, MO, USA). Cells were then harvested and surface stained for CD83-APC (BD Biosciences), CD1a-FITC, and E-cadherin-APC. Cells were fixed in 1% formaldehyde and analyzed on a BD FACS Canto II (BD Biosciences).

### Isolation and Stimulation of Primary LCs From Human Epidermis, Foreskin, and Papilloma Tissues

Deidentified surgical discards of abdominal skin or foreskin, or biopsies of respiratory papilloma tissue, were obtained within an hour of surgery. All tissues were washed in PBS containing antibiotics (100 μg/mL streptomycin and 100 U/mL of penicillin). For both abdominal skin and foreskin, adipose tissue was removed with forceps and a scalpel; small slices of the skin were placed in complete RPMI media plus 2.5 U/mL dispase (STEMCELL Technologies, Cambridge, MA, USA) with the epidermis facing up, and incubated overnight at 4°C. The next day, the epidermis was separated from the dermis using fine forceps and washed with PBS. To obtain single-cell preparations, abdominal and foreskin epidermis and papilloma biopsies were minced with a scalpel and scissors and then incubated with 0.05% trypsin (Gibco, Grand Island, NY, USA) and 100 U/mL DNAse I (Invitrogen, Thermo Fisher Scientific Inc.) at 37°C for 30 min. Complete RPMI media supplemented with 10% FBS was added to inactivate trypsin; cells were passed through a 70-μm cell strainer (Fisher) to obtain a single-cell suspension, collected by centrifugation at 600 *g* for 10 min, resuspended in PBS, and counted (Beckman Coulter, Indianapolis, IN, USA).

For the experiments shown in **Figure 4**, monocyte-derived iLCs and papilloma-derived LCs were isolated using Miltenyi beads for CD1a^+^, or CD207^+^ cells as described above, resuspended in medium without cytokines, and then stimulated for 4 h at 37°C with 10 ng/mL of recombinant active IL-36γ [amino acids (aa) 18–169] (R&D Systems), or with recombinant inactive full-length IL-36γ (aa 1–169) as a control.

For the experiments comparing LCs isolated from all three tissues, the LCs were stained with CD1a-FITC and CD207-PE, and sorted on a BD FACSAria as above. An aliquot of isolated LCs was immediately frozen for RNA isolation, and the remainder was cultured overnight in complete RPMI media supplemented with 100 ng/mL GMCSF and 10 ng/mL TGFβ1. The tissue-derived iLCs were then resuspended in fresh medium and stimulated for 4 h at 37°C with 1,500 ng/mL of poly(I:C) or 25 ng/mL of tumor necrosis factor α (TNFα) added to the medium. Addition of PBS was used as a control.

### Analysis of Expression of Proinflammatory and Anti-inflammatory Cytokines and Chemokines by Monocyte-Derived and Tissue-Derived iLCs

Cells were collected by centrifugation, and total mRNA isolated following DNase-1 treatment as per manufacturer's instructions (Qiagen, Valencia, CA, USA). The integrity of all mRNAs was determined by Bioanalyzer (RIN > 7.0) (Agilent Technologies, Santa Clara, CA, USA). To quantitate expression of the proinflammatory or anti-inflammatory cytokines/chemokines IL-1β, CCL-1, CCL-20, and TNFα, reverse transcription–polymerase chain reaction (RT-PCR) was performed using gene-specific intron-spanning primers, either purchased from Applied Biosystems/Life Technologies (Thermo Fisher Scientific, Waltham, MA, USA) or individually designed with a free bioinformatics program available on the Fisher website (Fisher Scientific [Thermo Fisher Scientific Inc., Pittsburg, PA, USA]), using the Human Universal Probe Library (Roche, Branford, CT, USA) for detection. Quantitative RT-PCR (qRT-PCR) was carried out on an Applied Biosystems 7900 HT (Applied Biosystems/Life Technologies [Thermo Fisher Scientific, Waltham, MA, USA]) following amplification with the iScript One-Step RT-PCR kit (Bio-Rad, Hercules, CA, USA). Total mRNA was reverse transcribed at 50°C for 10 min, followed by 5 min at 95°C to inactivate the reverse transcriptase and activate the Taq polymerase (hot-start), followed by 40 cycles of two-step PCR at 95°C for 15 s and 60°C for 1 min. Individual mRNA samples were run in duplicate, and the average Ct values for the gene of interest normalized to the average Ct values for the housekeeping gene (GAPDH) to calculate δ Ct. Results are expressed as level of expression relative to GAPDH, in order to compare relative expression of multiple cytokines and chemokines across different cells. Expression levels below that of GAPDH are shown as a decimal, and those above GAPDH expression are shown as whole numbers.

### Statistical Analysis

All results are expressed as mean ± S.D. Comparisons between two groups were done with a two-tailed, unpaired *t*-test. Comparisons between multiple groups were done with either a one-way analysis of variance (ANOVA) with Tukey-Kramer multiple-comparisons test or a nonparametric Kruskal-Wallis test with Dunn multiple-comparisons test when the standard deviations were markedly different. Significance was set at *p* < 0.05. For comparisons of tissue-derived iLCs, mean δ Cts ± SDs were determined, statistical significance was calculated by one-way ANOVA with Bonferroni multiple-comparisons correction, and results are expressed as numerical values relative to the level of GAPDH expression. For samples with no detectable mRNA, analysis was done, setting the δCt value to 10. Thus, the biological differences are likely to be underestimates.

## Results

### RRP Patients' Peripheral Blood Monocytes Generate Fewer iLCs Than Controls and Have a Different Distribution of Monocyte Subpopulations

**Figure 1:**
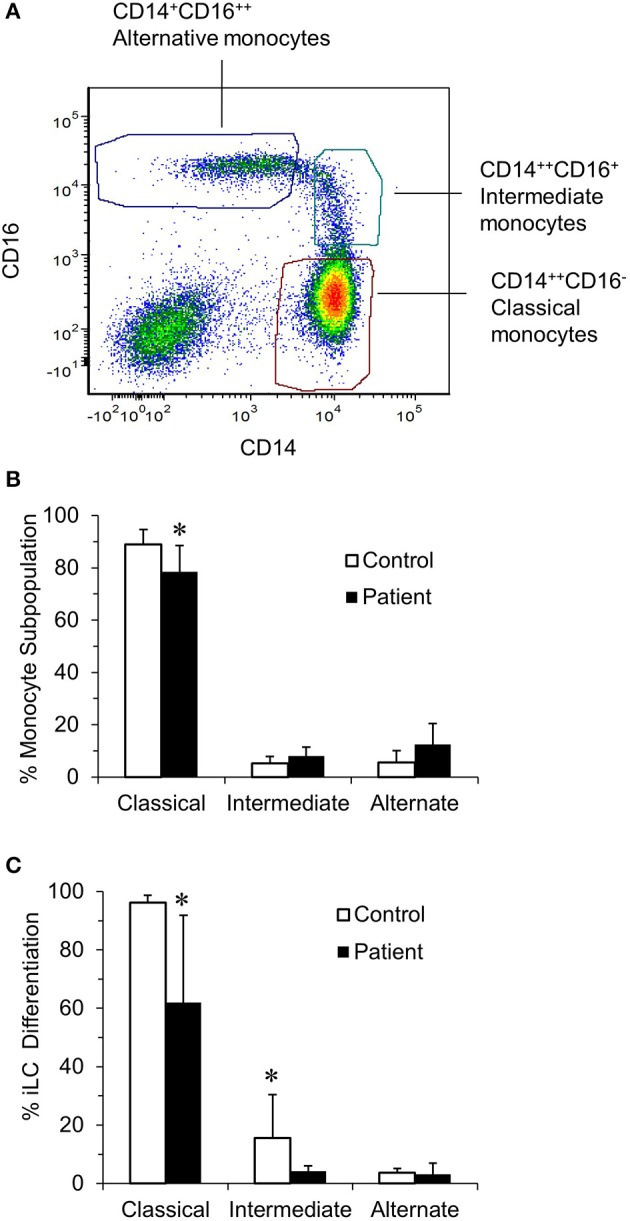
Identification of blood monocyte subsets in RRP patients and controls by flow cytometry. **(A)** Illustrates the characteristic of monocyte subsets from controls when immunostained with anti–CD14-PE and anti–CD16-Pacific Blue and sorted into CD14^++^CD16^−^ (classical), CD14^++^CD16^+^ (intermediate), and CD14^−^CD16^++^ (non-classical) subpopulations. **(B)** The percentage of classical, intermediate, and non-classical (alternate) monocytes in the controls (*n* = 8) and patients (*n* = 7) determined using a BD FACSAria cell sorter. There was a significant difference in the percentage of classical monocytes between patients and controls (*P* < 0.05). **(C)** Ability of monocyte subpopulations to generate iLCs; subpopulations of monocytes from control (*n* = 4) and patients (*n* = 6) were isolated and cultured for 7 days in complete RPMI medium supplemented with 100 ng/mL GMCSF, 20 ng/mL IL-4, and 10 ng/mL TGFβ1. On day 7, cells were harvested, surface stained with fluorochrome-labeled anti–CD1a-FITC and anti–E-cadherin-APC, and then analyzed by flow cytometry. There was a significant difference in the ability of classical monocytes from patients and controls to generate iLCs (*p* < 0.05). Results are shown as mean ± SD; data were analyzed by one-way ANOVA for **(B)** and two-tailed unpaired *t*-test for **(C)**. ^*^p < 0.05.

Langerhans cells can be generated from peripheral blood monocytes by incubating monocytes with IL-4, TGFβ1, and GMCSF (27). Peripheral blood mononuclear cell–derived monocytes can be separated into classical, intermediate, and non-classical subpopulations by gating on CD14 and CD16 expression (Figure 1A). While the number of PBMCs/mL of peripheral blood and the percentage of monocytes in the PBMC were comparable between patients and controls (data not shown), the relative percentage of monocyte subpopulations differed between patients and controls (Figure 1B). The percentage of classical monocytes (CD14^bright^, CD16^neg^) from patients was significantly lower than that from controls (88.96 ± 5.69 vs. 78.45 ± 10.01, *p* < 0.05). In contrast, the percentage of intermediate (CD14^bright^, CD16^dim^ cells) monocytes (5.28 ± 2.57 vs. 7.99 ± 3.37) and alternative (CD14^dim^, CD16^bright^) monocytes from controls and patients (5.5 ± 4.54 vs. 12.5 ± 7.92) were similar.

We then asked the potential of each monocyte subpopulation to differentiate into CD1a^+^, E-cadherin^+^ iLCs (Figure 1C). Almost all of the classical monocytes from controls differentiated into iLCs (96.5 ± 0.65%), fewer of the intermediate monocytes differentiated into iLCs (9.25 ± 2.21%), and very few of the alternative monocytes generated iLCs (2.75 ± 0.62%). While the patterns were very similar, significantly fewer of the classical monocytes from patients generated iLCs as compared to controls (61.6 ± 12.98%, *p* < 0.05), as did the intermediate monocytes (3.8 ± 0.8%, *p* < 0.05), while the difference with alternate monocytes was not significant due to the very small number of iLCs generated (3.0 ± 1.52%).

### Plasma PGE_2_ Expression Is Elevated in RRP Patients and Reduces Differentiation of Blood-Derived Monocytes Into iLCs

**Figure 2:**
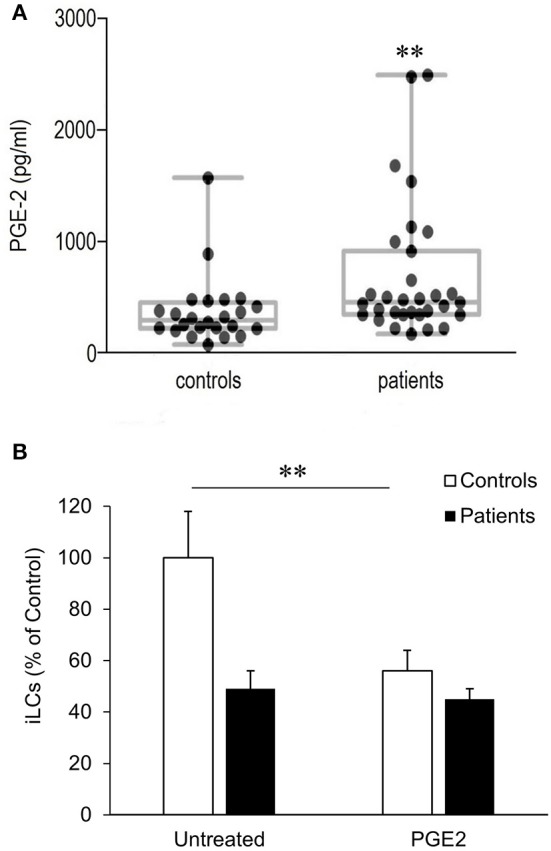
Plasma PGE_2_ levels and its effect on blood-derived monocytes differentiation to iLCs in patients with RRP and controls. **(A)** Plasma from RRP patients (*n* = 31) and controls (*n* = 24) were analyzed for PGE_2_ expression using competitive binding ELISA. Medians, upper and lower quartiles, and highest and lowest values are shown in the box plots. Both median and mean levels were significantly higher in patients (*p* < 0.015). **(B)** PGE_2_ exposure reduces differentiation of blood-derived monocytes iLCs; monocytes from patients (*n* = 16) and controls (*n* = 11) were incubated for 7 days in complete medium containing GMCSF, IL-4, and TGFβ1, in the presence or absence of 5 mM PGE_2_. On day 7, CD1a^+^ CD207^+^ iLCs were positively selected by magnetic beads and counted. Results are expressed as mean ± SD relative to the baseline level of differentiation of untreated monocytes from controls. There was a highly significant reduction in the differentiation of control monocytes with PGE_2_ exposure (*p* < 0.01), but not patient monocytes. Data were analyzed by two-tailed *t*-test. ^**^p < 0.01.

Previously, we showed that COX-2 is constitutively overexpressed in respiratory papillomas and the adjacent upper airway epithelium of RRP patients, leading to an increased synthesis of PGE_2_ in these tissues (24). We have now found that the mean concentration of PGE_2_ in the plasma of patients (690 ± 109 pg/mL) is also significantly higher than in plasma obtained from controls (372 ± 63 pg/mL) (*p* < 0.015) (Figure 2A). In addition, there was greater variability in plasma PGE_2_ levels in patients compared with controls. Of note, the plasma PGE_2_ levels in all patients (*N* = 6) studied in Figure 1C were above the mean value for both patients and controls. In addition, the patient with the lowest number of classical monocytes had the highest plasma level of PGE_2_. Prostaglandin E_2_ levels did not correlate with the severity of disease, calculated at the time of surgery for the RRP patients.

We therefore asked whether the higher PGE_2_ plasma levels in patients with RRP might contribute to the reduced differentiation of their monocytes into iLCs, determining the effect of added PGE_2_ on differentiation (Figure 2B). Monocytes from eight additional controls were compared to monocytes from three additional patients with high PGE_2_ plasma levels (2,640, 1,560, and 1,090 pg/mL). Prostaglandin E_2_ added to control monocytes suppressed their ability to differentiate into iLCs from 31.1 ± 4.52% to 17.9 ± 2.62% (*p* < 0.007). The patients' monocytes showed lower levels of iLC differentiation in the absence of added PGE_2_ (Figure 2B), and differentiation did not decrease further when exogenous PGE_2_ was added (16.4 ± 2.76% vs. 14.3 ± 1.76%, *p* < 0.76), suggesting that they were already maximally repressed.

### PGE_2_ Alters the CCL1/CCL20 Ratio Expression of Control iLCs

We previously showed that day 7 monocyte cultures enriched with iLCs from controls expressed moderately high levels of *CCL1* mRNA at baseline, which was further increased following IL-36γ stimulation (27). This inflammatory chemokine selectively binds CCR8 present on monocytes, macrophages, neutrophils, T_H_2 T cells, Tregs, and other cells (32, 33). In contrast, the iLCs from RRP patients showed decreased levels of baseline *CCL1* mRNA expression, which correlated with RRP disease severity. In contrast, the iLCs from patients showed increased baseline expression of the more anti-inflammatory T_H_2-like chemokine *CCL20* (27). We therefore asked whether PGE_2_ altered the baseline mRNA expression of *CCL1* and/or *CCL20* by monocyte-derived iLCs. When control iLCs (*n* = 7) were pre-incubated with PGE_2_, there was a trend toward a reduction in *CCL1* mRNA expression and an increase in *CCL20* mRNA expression. Although the change in CCL1 or CCL20 did not reach significance alone, the *CCL1/CCL20* mRNA ratio for the control iLCs exposed to PGE_2_ was significantly reduced (*p* < 0.02) (Supplementary Figure 1A). In contrast, the patients' CCL1/CCL20 expression ratio, which was low because CCL1 mRNA expression is poorly expressed by RRP patients' iLCs, did not change significantly in response to the addition of PGE_2_ (Supplementary Figure 1B). This lack of response to PGE_2_ is consistent with the observation that added PGE_2_ did not suppress differentiation of monocytes from RRP patients to iLCs (Figure 2).

### PGE_2_ and IL-36γ Induce Maturation of Monocyte-Derived iLCs

**Figure 3:**
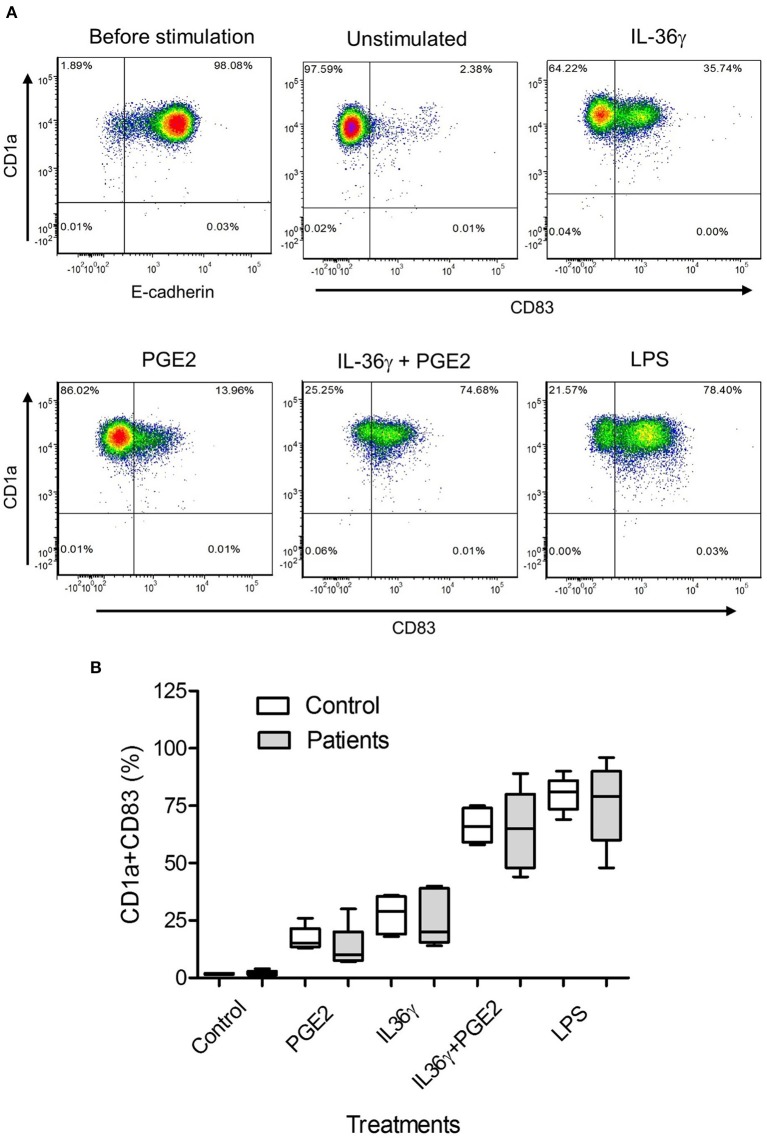
Prostaglandin E_2_ and IL-36γ induce maturation of monocyte-derived iLCs. **(A)** Representative experiment of purified monocytes from a patient with RRP treated with 250 nM PGE_2_, 10 ng/mL of IL-36γ, PGE_2_+IL-36γ, or 100 ng/mL LPS for 48 h and then stained for CD1a, E-cadherin, and the maturation marker CD83 and analyzed by flow cytometry. **(B)** Purified monocyte-derived iLCs from controls (*n* = 5) and patients (*n* = 5) were cultured with 250 nM PGE_2_, 10 ng/mL of IL-36γ, PGE_2_+IL-36γ, or 100 ng/mL LPS for 48 h and then similarly stained and analyzed. Treatment of iLCs with either the combination of PGE_2_ + IL-36γ or with LPS induced significantly more maturation than either PGE_2_ or IL-36γ alone. There was no significant difference between patients and controls with any of the treatments. Results are shown as mean ± SD, analyzed by Kruskal-Wallis test with Dunn multiple-comparisons correction.

To determine whether PGE_2_ also affected iLC maturation, identified by CD83 expression (34), we exposed monocyte-derived iLCs from patients and controls to PGE_2_, IL-36γ (a proinflammatory cytokine constitutively expressed by papilloma cells) (28), a combination of these mediators, or LPS as a positive control. A representative flow analysis is shown in Figure 3A. Prostaglandin E_2_ and IL-36γ both induced modest numbers of iLCs to express CD83. However, the combination of IL-36γ and PGE_2_ strongly induced almost all of the iLCs to express CD83, similar to the effect of LPS. There was no difference between patients and controls in iLC maturation in response to any of these mediators (Figure 3B), but there was significant variation in CD83 expression by maturing iLCs from patient-derived LCs compared with controls under all culture conditions. Thus, unlike the suppressive effects of PGE_2_ on control monocyte iLC differentiation, PGE_2_, IL-36γ, and their combination did not alter iLC maturation once differentiation was completed.

### CCL1 and CCL20 Expression by RRP Patients' Monocyte-Derived iLCs Is Altered in Response to IL-36γ

**Figure 4:**
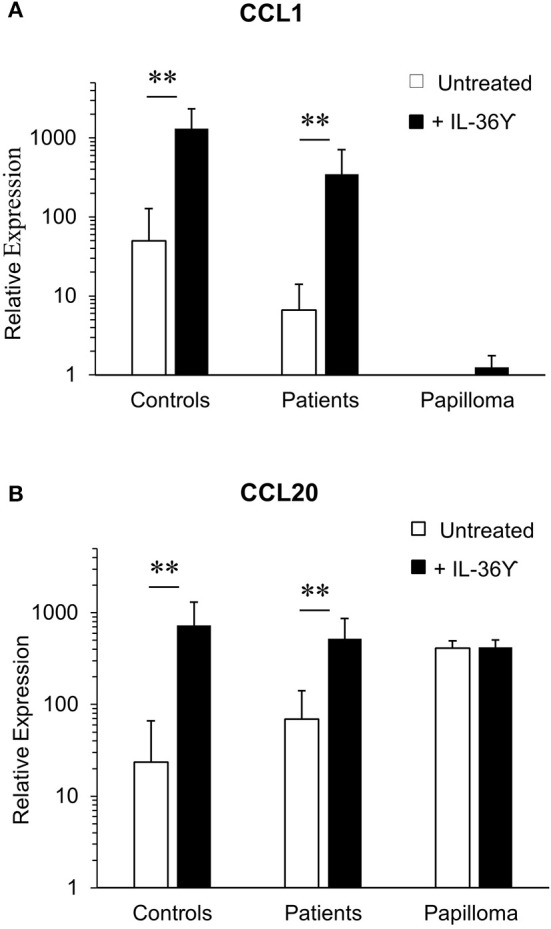
IL-36γ stimulation increases expression of CCL-1 and CCL-20 mRNA by monocyte-derived iLCs, but not iLCs isolated from papilloma tissues. Monocyte-derived iLCs from controls (*n* = 8) and patients (*n* = 14), and iLCs isolated from papilloma biopsy samples (*n* = 4), were suspended in medium without cytokines and stimulated for 4 h at 37°C with 10 ng/mL of recombinant active IL-36γ or with the recombinant inactive full-length IL-36γ as a control (untreated). Total mRNA was isolated, and expression of **(A)** CCL1 and **(B)** CCL20 analyzed by qRT-PCR. IL-36γ significantly enhanced expression of both chemokines by monocyte-derived iLCs from both patients and controls relative to untreated iLCs, but had no effect on papilloma-derived iLCs (*p* < 0.01). Results are expressed as mean ± SD relative to the very low level of CCL1 expression by the papilloma-derived iLCs, in order to compare across both cell types and conditions. Data were analyzed by Mann-Whitney *U*-test. ^**^p < 0.01.

We then asked whether expression of *CCL1* and *CCL20* mRNA by purified monocyte-derived iLCs from controls and patients differed from iLCs isolated from papilloma tissues (Figure 4). As previously reported (15), relative baseline *CCL1* mRNA expression by monocyte-derived iLCs from controls (50.7 ± 27.7) was significantly higher than that from patients (6.31 ± 1.95), and both types of cells increased expression after IL-36γ treatment (controls 1,164 ± 319, patients 348 ± 97, *p* < 0.01) (Figure 4A). In contrast, iLCs from papillomas showed little, if any, *CCL1* mRNA expression at baseline and very little increase after IL-36γ treatment. Baseline expression of *CCL20* mRNA (Figure 4B) by monocyte-derived iLCs from controls (24.17 ± 15) and patients (68.9 ± 19.1) was significantly increased after IL-36γ treatment (controls 731 ± 206; patients (519 ± 92) (controls *p* < 0.01; patients *p* < 0.01). Unlike *CCL1*, papilloma-derived iLCs expressed very high levels of *CCL20* mRNA at baseline that did not further increase following IL-36γ stimulation (410 ± 65 and 422 ± 80, respectively).

### Papilloma-Derived iLCs Express *CCL1* mRNA After Removal From Their Tissue Microenvironment and Constitutively Express *IL-36γ* mRNA

Finally, we asked whether papilloma-resident iLCs also differed from monocyte-derived iLC in their expression of other cytokines or chemokines in response to IL-36γ. However, repeated tries were unable to induce any measurable responses to either IL-36γ or to the related proinflammatory cytokine IL-1β. We therefore considered the possibility that iLCs were refractory to stimulation because of the immunosuppressive environment within papilloma tissues. To test this, we removed the iLCs from the papillomas and incubated them overnight in supporting medium containing GMCSF and TGFβ1. Then, we stimulated them with two strong iLC stimuli, the TLR3 agonist poly (I:C) or TNFα (28), and measured expression of several cytokines and chemokines, including *IL-36*γ mRNA, because we had previously shown that IL-36γ stimulation upregulates its own mRNA expression through a positive feedback loop in monocyte-derived iLCs and keratinocytes (27, 28). Other mucosal and skin-derived iLCs are of an embryonic origin, not monocytes (35–38), so we used iLCs isolated from surgical discards of abdominal skin and foreskin for comparison to the papilloma iLCs.

**Figure 5:**
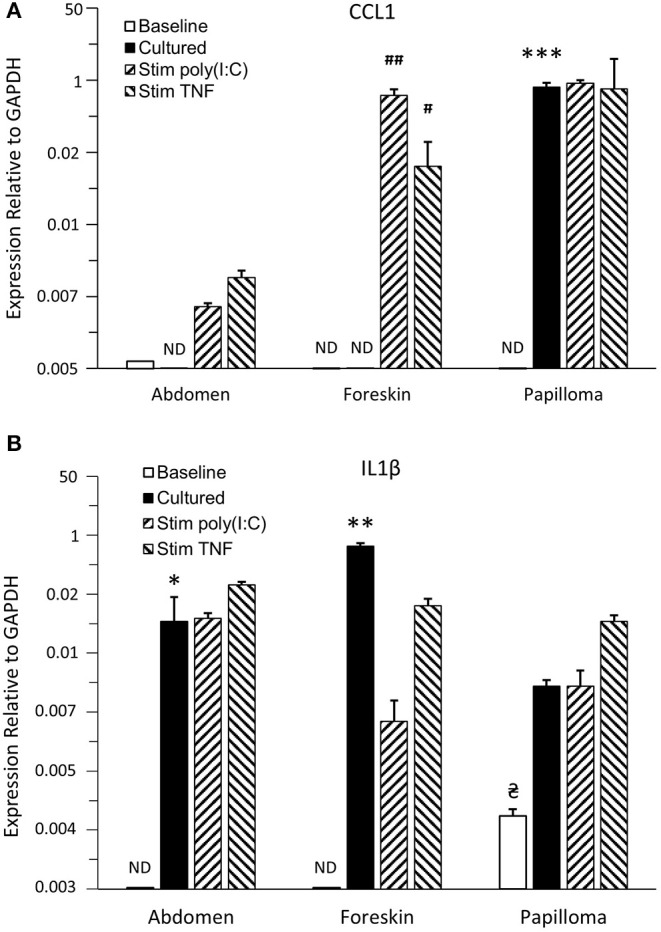
Expression of proinflammatory cytokines/chemokines by iLCs from respiratory papillomas, abdominal skin, and foreskin. Langerhans cells were isolated from papilloma biopsies (*n* = 4–5, depending on the condition analyzed), from normal abdominal skin (*n* = 3–4) and normal foreskin tissues (*n* = 3–4). Expressions of CCL1 **(A)** and IL-36γ **(B)** were measured by qRT-PCR in iLCs immediately after isolation (baseline) or after overnight culture in medium supplemented with 100 ng/mL GMCSF and 10 ng/mL TGFβ1 followed by addition of PBS (cultured), or stimulation with 1,500 ng/mL of poly(I:C) or 25 ng/mL of TNFα for 4 h to determine the effects of removal from their micromilieu and ability to be stimulated by proinflammatory cytokines. Results are mean ± SD, normalized to GAPDH, and analyzed by ANOVA. ^*^p < 0.05 compared to baseline, ^**^p < 0.01 compared to baseline expression, ^***^p < 0.001 compared to baseline, #*p* < 0.05 compared to cultured iLCs treated with PBS, ##*p* < 0.01 compared to cultured iLCs treated with PBS, 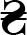 *p* < 0.05 compared to abdomen and foreskin baseline levels. ND = not detectable. Note that CCL1 was significantly upregulated only when iLCs from papilloma tissue were removed from their immunosuppressive environment and that only papilloma iLCs expressed IL-36γ at baseline, but all iLCs showed elevated *IL-36*γ expression when cultured away from the tissue. Responses to cytokine stimulation were variable between iLCs from the three tissues.

Immature LCs from papillomas and foreskin did not express detectable *CCL1* mRNA expression at baseline, and abdominal skin iLCs expressed extremely low levels of CCL1 (Figure 5A). Overnight culture alone had no significant effect on expression by abdominal or foreskin iLC, but papilloma iLCs markedly upregulated *CCL1* mRNA, approaching the expression levels of *GAPDH* (>200-fold increase compared to baseline levels, *p* < 0.001). These results suggest that iLCs from the papillomas appear “primed” to express CCL1 but are suppressed when in the immunosuppressive papilloma microenvironment (15, 27). Subsequent stimulation of papilloma iLCs with poly(I:C) or TNFα did not further increase their high level of *CCL1* mRNA expression. Foreskin-derived iLCs expressed significant *CCL1* after poly (I:C) and TNFα stimulation [97.9-fold (*p* < 0.01) and 17.2-fold (*p* < 0.05) respectively], whereas increases in CCL1 expression by abdominal skin iLCs after stimulation were not significant.

Immature LCs from papillomas expressed detectable levels of *IL-36*γ mRNA at the time of isolation from the tissues (Figure 5B), which was significantly different from the baseline expression by either abdomen or foreskin iLCs (*p* < 0.05) and might reflect the fact that they were exposed to IL-36γ peptide *in vivo*. Expression was further increased after overnight culture outside of papilloma tissue, although this increase was not significant because of the small number of samples. Immature LCs from the abdomen and foreskin expressed no *IL-36*γ mRNA at baseline, but did also upregulate expression following culture in the absence of their normal tissue microenvironments (14.1- and 114-fold, *p* < 0.05 and *p* < 0.01, respectively). None of the cells showed further significant increases with poly (I:C) or TNFα treatment.

The pattern of expression of the other cytokines/chemokines analyzed was quite similar for iLCs from all three tissues and generally quite high under all conditions (Supplementary Figure 2). Immature LCs from the papillomas and foreskin expressed high levels of *CCL20* mRNA that were close to *GAPDH* levels at baseline, with no significant change after removal from their microenvironments or following stimulation with either poly(I:C) or TNFα (Supplementary Figure 2A). Abdominal iLCs made less *CCL20* mRNA at baseline, but it was upregulated 27-fold (*p* < 0.01) to the levels of the other iLCs after removal from the tissue, with no further significant increase after stimulation. Immature LCs from the three different tissues also expressed high levels of *IL-1*β mRNA and TNFα under all conditions (Supplementary Figures 2B,C). It was notable that iLCs from papillomas expressed significantly more IL-1β at baseline than the abdomen and foreskin iLCs (52- and 8-fold, respectively, *p* < 0.05), but this dropped back down to the levels seen with the other cells after overnight culture (Supplementary Figure 2B). This high level of IL-1β message may not be of functional significance because posttranslational processing is required for the production of active IL-1β (39, 40).

## Discussion

We have shown alterations in both monocyte- and tissue-derived iLCs from patients with RRP and postulate that these alterations could contribute to the anti-inflammatory immune response these patients make to persistent HPV6/11 infection (8, 9). Monocytes can be subdivided into three main subgroups, based on their surface markers (41). The classical monocyte subpopulation is the major source of monocyte-derived iLCs (41), although we have now found that the intermediate and alternate subpopulations can also generate small numbers of iLCs. The reduced yield of iLCs from patients' PBMCs that we observed may be explained, in part, by the reduction in the percentage of classical monocytes, but the efficiency of differentiation into iLCs is also impaired when starting with equal numbers of classical monocytes. One mechanism for this impairment may be the exposure of the monocytes to PGE_2_ *in vivo*. We had previously reported that PGE_2_ was elevated in the airway tissues of RRP patients (24) and have now found that their plasma concentrations of PGE_2_ are also increased. The suppressive effect of PGE_2_ on monocyte differentiation into iLCs must not be easily reversible because the patients' monocytes do not recover during the time they are in culture and differentiating into iLCs, and they are refractory to further inhibition of differentiation by treatment with PGE_2_. This suggests that they are already “locked” into this lower iLC differentiation state.

Chronic exposure to PGE_2_ *in vivo*, however, does not explain why papilloma tissues contain an abundant number of iLCs that are not activated (8, 27). The tissue iLCs are exposed to both IL-36γ and to PGE_2_ (9, 26–28). We have now shown that either PGE_2_ or the active form of IL-36γ is sufficient to partially induce activation of monocyte-derived iLCs from both patients and controls, and the combination is as effective as LPS in inducing CD83 expression. Thus, papilloma LCs would be expected to be activated and leave the tissue, migrating to lymph nodes where they would directly or indirectly present HPV antigens and stimulate effector T cells (42–44). However, this does not occur in papillomas. Several explanations for this paradox can be considered. (1) There may be insufficient extracellular concentrations of the active form of IL-36γ in papilloma tissues to induce activation. We have previously shown that IL-36γ is poorly released from HPV-infected cells (27). Moreover, the precursor IL-36γ molecule must be processed to generate the active proinflammatory cytokine. This happens via extracellular proteolysis by proteases such as neutrophil elastase (45), and papilloma tissue is devoid of activated neutrophils (8). (2) The functionally active Tregs in the papillomas (15) may suppress iLC activation, leading to a positive feedback loop between iLCs and Tregs that maintains Treg stability and retains iLC immaturity (43). (3) Tissue-resident iLCs may respond differently to activation stimuli than monocyte-derived iLCs, and few if any monocyte-derived iLCs may be in these premalignant tumors. The iLCs in normal, non-inflamed epithelia are not bone marrow derived (37, 46), although to our knowledge, no one has identified the source of laryngeal iLCs. Immature LC precursors migrate to epithelial surfaces early in embryogenesis (35, 36, 38, 46), becoming long-lived iLCs that exist in direct contact with keratinocytes through tight junctions, and they show some differences from monocyte-derived iLCs (41, 47, 48). Monocytes can enter inflamed/damaged tissues, or tumors, and differentiate into iLCs (48), but there is no evidence of inflammation in respiratory papillomas (8, 12, 49). However, we cannot exclude the possibility that the patients' iLCs are similar to iLCs of systemic LC histiocytosis, further justifying the use of monocyte derived iLCs in this study (50). The reason(s) for the failure of papilloma iLCs to be constitutively activated remains to be determined. However, it is possible that the functional Tregs present in papillomas (15) block the abundant iLCs in these lesions from becoming activated and matured and their migration from the papilloma epithelium to regional lymph nodes where they would transfer HPV antigens to myeloid DCs, or present them directly to T cells (41).

We previously reported that monocyte-derived iLCs from RRP patients with severe disease express much lower baseline levels of *CCL1* mRNA than iLCs from patients with mild/moderate disease or controls (27). We now showed that baseline *CCL1* expression by papilloma-derived iLCs is almost undetectable. However, culturing these cells overnight outside of the immunosuppressive micromilieu present in papillomas releases these cells to express high levels of CCL1 mRNA. Expression of CCL1 by iLCs in tissues would support the influx of neutrophils, macrophages, monocytes, T_H_2-like T cells, Tregs, and other cells (32, 33), while CCL20 (51) that is abundantly expressed in papillomas (9) would sustain these lesions. CCL1 can also activate and mobilize tissue-derived iLCs (52, 53). Absence of CCL1 expression by iLCs in papillomas may be caused by the abundant functional Tregs in these lesions (15). Thus, targeting Tregs may be an effective therapeutic approach to raise “functional” CCL1 levels. Breaking the Treg-iLC interdependence that stabilizes Treg function and maintains iLC immaturity (43) may help reverse the immunosuppressive T_H_2-like/Treg adaptive micromilieu present in theses tissues, because iLCs in papillomas can make CCL1 spontaneously when removed from their *in vivo* anti-inflammatory microenvironment. Immature LCs from the control abdominal skin and foreskin expressed low levels of *CCL1* at baseline that did not increase when cultured overnight, consistent with the fact that these are from non-inflamed healthy tissues.

We previously reported that cultured monocyte-derived iLCs expressed *IL-36*γ mRNA and that it was upregulated by a positive feedback loop when the cells were stimulated with activated IL-36γ (27). In the present study, only papilloma-derived iLCs expressed measurable *IL*-36γ mRNA at baseline, suggesting that the levels of extracellular IL-36γ in the papilloma tissue might be sufficient to activate the iLC IL-36γ feedback loop (27) *in vivo* and that papilloma iLCs can respond even if they cannot be induced to mature.

Immature LCs from all three types of tissue expressed relatively high levels of *CCL20, IL-1*β, and *TNF*α mRNA at baseline, so they are not immunologically inert. The general patterns of expression were similar across the iLCs from the three tissues for the most part, but there were some variations in robustness of expression in response to different stimulating stimuli. These results are reminiscent of our recent report from organotypic cultures of keratinocytes obtained from different anatomical sites that show marked differences in the expression of immune-response genes/gene pathways both pre- and post-HPV16 transduction (54). This suggests that keratinocyte-iLC “innate unit” signaling is likely to be site-specific, orchestrated by the origin of the keratinocytes and iLCs and likely modulated by the microbiome at that given site (54, 55). We are presently exploring this possibility.

In summary, there are clear differences in the innate immune system in RRP patients that underlie the polarized T_H_2-/Treg HPV-specific responses in papillomas and supports persistent HPV6/11 infection. What remains elusive is the cause(s) of these innate and adaptive differences. It is intriguing to consider RRP, and for that matter other HPV-related diseases such as cervical and head and neck cancers, as potential “microbe selective” primary immunodeficiency diseases. This would be much like other primary immune deficiencies that show restricted microbe/microbial family vulnerability (56). Cross-sectional and vertical genetic studies have thus far failed to identify primary DNA sequence single nucleotide polymorphisms that define a predisposition to develop RRP or the severity of this disease. However, we have reported the enrichment of select class II genotypes and restricted KIR gene/haplotypes (13, 57). Future studies directed at whole-genome sequencing or epigenetic gene regulation may ultimately shed light on why only a very small fraction of individuals develop RRP or HPV-induced cancers.

## Data Availability Statement

The datasets generated for this study are available on request to the corresponding author.

## Ethics Statement

The studies involving human participants were reviewed and approved by Northwell Health IRB protocol 13-526B. The patients/participants provided their written informed consent to participate in this study.

## Author Contributions

MI, JD, and FL contributed to the design of the study, conduct of the experiments, analysis of the data, and preparation and editing of the manuscript. AA contributed to the design of the study, review of the data, and review of the manuscript. BS and VB contributed to the design of the study, analysis of the data, and preparation and editing of the manuscript.

### Conflict of Interest

The authors declare that the research was conducted in the absence of any commercial or financial relationships that could be construed as a potential conflict of interest.

## Supplementary Material

Supplementary Figure 1 Monocytes from controls (*n* = 7) and patients (*n* = 3) were differentiated into iLCs in the presence or absence of 250 mM PGE_2_ and their expression of CCL1 and CCL20 mRNA identified by quantitative PCR. Control monocytes exposed to PGE_2_ expressed a significantly lower (*p* < 0.02) mRNA CCL1/CCL20 ratio than the same monocytes untreated with PGE_2_ **(A)**. The CCL1/CCL20 ratio for patients with RRP which was low because of the markedly reduced CCL1 expression did not change with the addition of PGE_2_ **(B)**.

Supplementary Figure 2 Expression of cytokines/chemokines by iLCs from respiratory papillomas, abdominal skin, and foreskin. Langerhans cells were isolated from papilloma biopsies (*n* = 4–5, depending on the condition analyzed), from normal abdominal skin (*n* = 3–4) and normal foreskin tissues (*n* = 3–4). Expression of CCL20 **(A)**, IL1β **(B)**, and TNFα **(C)** was measured by qRT-PCR in iLCs immediately after isolation (baseline), or after overnight culture in medium supplemented with 100 ng/mL GMCSF and 10 ng/mL TGFβ1 followed by addition of PBS (cultured) or stimulation with 1,500 ng/mL of poly(I:C) or 25 ng/mL of TNFα for 4 h to determine the effects of removal from their micromilieu and ability to be stimulated by proinflammatory cytokines. Results are mean ± SD, normalized to GAPDH, and analyzed by Anova. ^*^*p* < 0.05 compared to baseline, ^**^*p* < 0.01 compared to baseline,  *p* < 0.5 compared to abdomen and foreskin baseline levels. Note that baseline IL1β was significantly upregulated compared to baseline levels in abdomen and foreskin iLCs.

Supplementary Table 1 Demographics and disease severity of patients with RRP.
