# Supplementary figures and images for: Altered Monocyte and Langerhans Cell Innate Immunity in Patients With Recurrent Respiratory Papillomatosis (RRP)
Source: Front Immunol. 2020 Mar 10;11:336. doi: 10.3389/fimmu.2020.00336 (PMC7076114; doi:10.3389/fimmu.2020.00336)

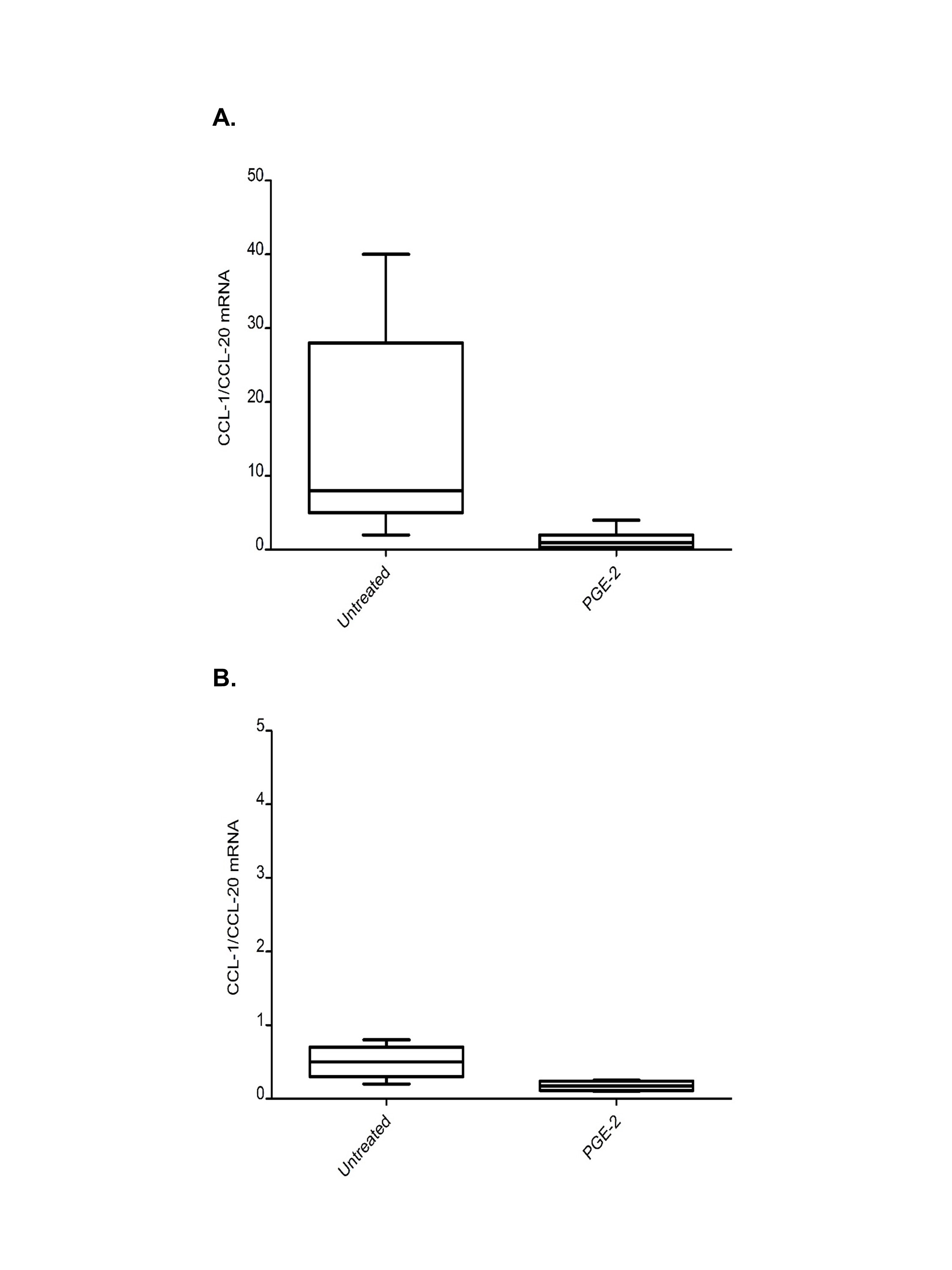

Supplement: Supplementary Figure 1 — Monocytes from controls (n = 7) and patients (n = 3) were differentiated into iLCs in the presence or absence of 250 mM PGE2 and their expression of CCL1 and CCL20 mRNA identified by quantitative PCR. Control monocytes exposed to PGE2 expressed a significantly lower (p < 0.02) mRNA CCL1/CCL20 ratio than the same monocytes untreated with PGE2 (A). The CCL1/CCL20 ratio for patients with RRP which was low because of the markedly reduced CCL1 expression did not change with the addition of PGE2 (B). [file Image_1.JPEG]

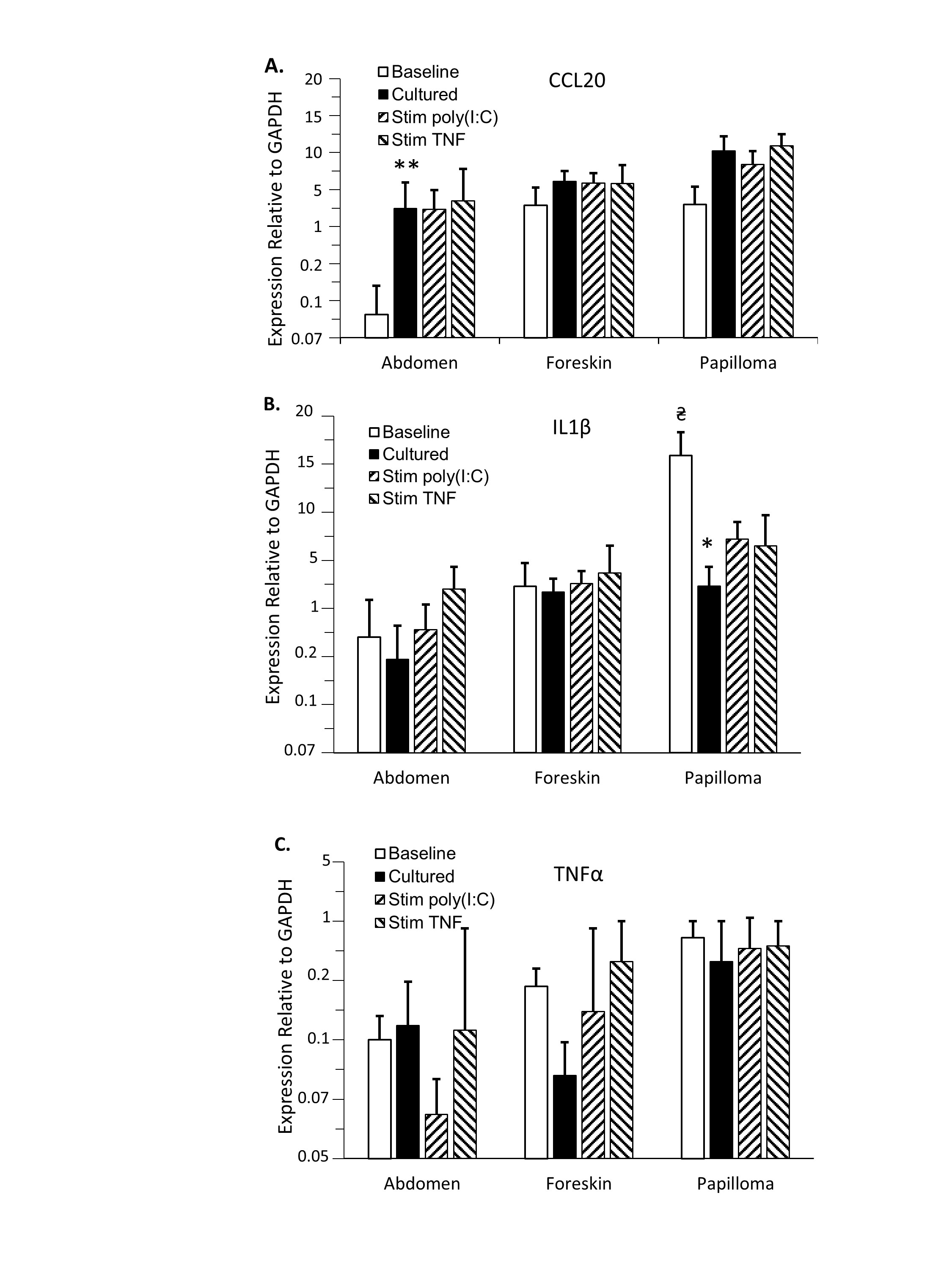

Supplement: Supplementary Figure 2 — Expression of cytokines/chemokines by iLCs from respiratory papillomas, abdominal skin, and foreskin. Langerhans cells were isolated from papilloma biopsies (n = 4–5, depending on the condition analyzed), from normal abdominal skin (n = 3–4) and normal foreskin tissues (n = 3–4). Expression of CCL20 (A), IL1β (B), and TNFα (C) was measured by qRT-PCR in iLCs immediately after isolation (baseline), or after overnight culture in medium supplemented with 100 ng/mL GMCSF and 10 ng/mL TGFβ1 followed by addition of PBS (cultured) or stimulation with 1,500 ng/mL of poly(I:C) or 25 ng/mL of TNFα for 4 h to determine the effects of removal from their micromilieu and ability to be stimulated by proinflammatory cytokines. Results are mean ± SD, normalized to GAPDH, and analyzed by Anova. *p < 0.05 compared to baseline, **p < 0.01 compared to baseline, p < 0.5 compared to abdomen and foreskin baseline levels. Note that baseline IL1β was significantly upregulated compared to baseline levels in abdomen and foreskin iLCs. [file Image_2.JPEG]
