# Supplementary material for: Altered Monocyte and Langerhans Cell Innate Immunity in Patients With Recurrent Respiratory Papillomatosis (RRP)
Source: Front Immunol. 2020 Mar 10;11:336. doi: 10.3389/fimmu.2020.00336 (PMC7076114; doi:10.3389/fimmu.2020.00336)
Supplement: Supplementary Table 1 — Demographics and disease severity of patients with RRP. [file Table_1.DOCX]

| **Supplement Table 1. Demographics and disease severity of patients with RRP.** |
| --- |

| Patient # | Age | Sex | Severity Score |
| --- | --- | --- | --- |
| \| 1 \| \| --- \| \| 2 \| \| 3 \| \| 4 \| \| 5 \| \| 6 \| \| 7 \| \| 8 \| \| 9 \| \| 10 \| \| 11 \| \| 12 \| \| 13 \| \| 14 \| \| 15 \| \| 16 \| \| 17 \| \| 18 \| \| 19 \| \| 20 \| \| 21 \| \| 22 \| \| 23 \| \| 24 \| \| 25 \| \| 26 \| \| 27 \| \| 28 \| \| 29 \| \| 30 \| \| 31 \| \| 32 \| \| 33 \| \| 34 \| \| 35 \| \| 36 \| | \| 49 \| \| --- \| \| 63 \| \| 71 \| \| 76 \| \| 65 \| \| 64 \| \| 30 \| \| 37 \| \| 33 \| \| 57 \| \| 65 \| \| 31 \| \| 58 \| \| 63 \| \| 67 \| \| 49 \| \| 50 \| \| 41 \| \| 54 \| \| 68 \| \| 62 \| \| 72 \| \| 63 \| \| 16 \| \| 56 \| \| 77 \| \| 77 \| \| 23 \| \| 37 \| \| 15 \| \| 23 \| \| 44 \| \| 64 \| \| 34 \| \| 89 \| \| 75 \| | \| M \| \| --- \| \| F \| \| M \| \| M \| \| M \| \| M \| \| M \| \| F \| \| F \| \| M \| \| M \| \| F \| \| M \| \| M \| \| M \| \| F \| \| M \| \| M \| \| M \| \| F \| \| M \| \| M \| \| F \| \| M \| \| M \| \| M \| \| F \| \| M \| \| M \| \| M \| \| M \| \| M \| \| M \| \| F \| \| F \| \| F \| | \| 0.099 \| \| --- \| \| 0.109 \| \| 0.068 \| \| 0.014 \| \| 0.121 \| \| 0.006 \| \| 0.157 \| \| 0.071 \| \| 0.019 \| \| 0.007 \| \| 0.123 \| \| 0.238 \| \| 0.007 \| \| 0.009 \| \| 0.102 \| \| 0.011 \| \| 0.001 \| \| 0.092 \| \| 0.003 \| \| 0.022 \| \| 0.023 \| \| 0.022 \| \| 0.001 \| \| 0.075 \| \| 0.002 \| \| 0.011 \| \| 0.026 \| \| 0.067 \| \| 0.004 \| \| 0.016 \| \| 0.056 \| \| 0.004 \| \| 0.013 \| \| 0.018 \| \| 0.025 \| \| 0.008 \| |
